# Orbital Intravascular Natural Killer/T-cell Lymphoma: An Unusual Cause of Ocular Symptoms

**DOI:** 10.31662/jmaj.2022-0063

**Published:** 2022-06-17

**Authors:** Naohiro Uchio, Daiki Yashita, Akihito Hao, Atsuhito Nakayama, Shigeki Morita, Tsuyoshi Takahashi, Masaya Mori, Hideyuki Matsumoto

**Affiliations:** 1 Department of Neurology, Mitsui Memorial Hospital, Tokyo, Japan; 2 Department of Pathology, Mitsui Memorial Hospital, Tokyo, Japan; 3 Department of Hematology, Mitsui Memorial Hospital, Tokyo, Japan

**Keywords:** intravascular lymphoma, natural killer/T-cell lymphoma, extranodal non-Hodgkin lymphoma, autopsy

## Abstract

Orbital intravascular lymphoma is rare and typically of B-cell lineage. In this study, we report a patient who developed orbital lesions of intravascular natural killer/T-cell lymphoma (IVNKL), an extremely rare lymphoma. An 88-year-old man presented with rapidly progressive right vision loss and double vision. A neurological examination revealed that he had decreased visual acuity and severe oculomotor impairment in the right eye. Magnetic resonance imaging showed right-dominant, nonmass lesions in both orbits. No lesions were found in the lymph nodes, skin, or brain. The patient received immunosuppressive and antifungal therapy, but his clinical condition rapidly deteriorated, and he died of multiple organ failure. Autopsy revealed natural killer/T-cell lymphoma proliferation within the lumina of small blood vessels in multiple organs, including the ocular adnexa of the right orbit. These findings show that he was ultimately diagnosed with IVNKL. IVNKL could initially cause ocular symptoms due to the involvement of the ocular adnexa. Ocular involvements have not been described previously. Even if patients initially present with only ocular symptoms, IVNKL should be considered.

## Introduction

Lymphoma accounts for 6%-8% of all orbital tumors ^(1)^. Although different histologic types of orbital lymphoma have been described, intravascular lymphoma is rare, with only cases of B-cell lineage documented ^(2)^. In this study, we report a patient who initially developed orbital lesions of intravascular natural killer/T-cell lymphoma (IVNKL), an extremely rare lymphoma ^(3)^, whose diagnosis was later confirmed via autopsy.

## Case Report

**Figure 1.**
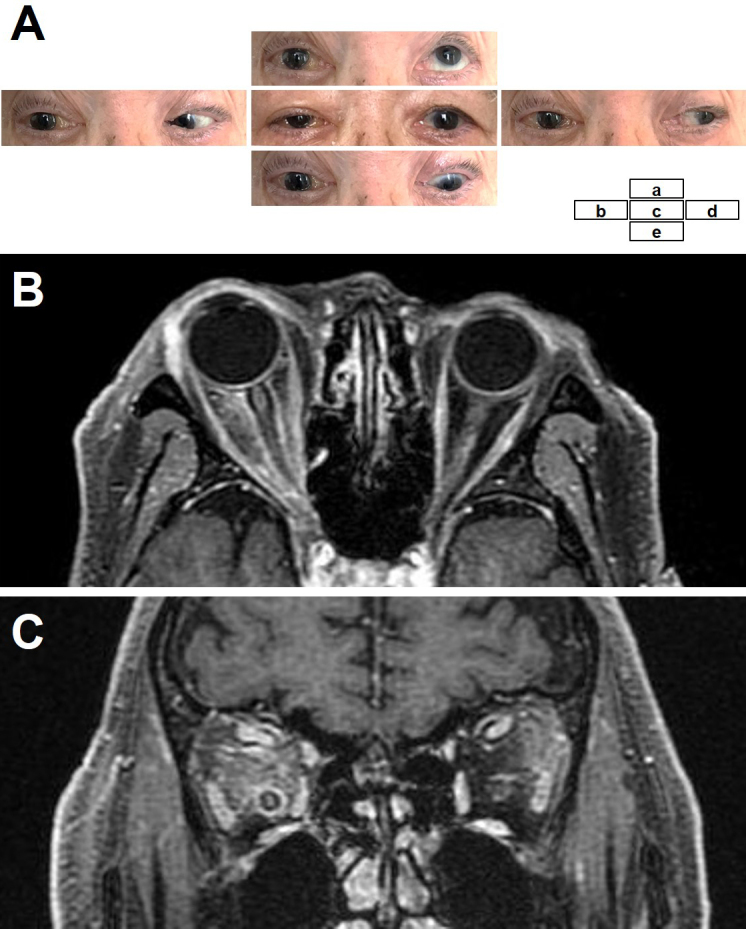
Clinical and magnetic resonance imaging findings. (A) Oculomotor impairment of the right eye: upward (a), right (b), forward (c), left (d), and downward (e) gazes. Right-dominant, non-mass-like enhancements in the adipose tissues in both orbits in axial (B) and coronal (C) sections of gadolinium-enhanced T1-weighted imaging.

An 88-year-old man presented with right vision loss and double vision after a weight loss of 4 kg in 3 months and mild fever for 1 month. He had no skin symptoms or lymph node swelling. Neurological examination showed decreased visual acuity (counting fingers) and severe oculomotor impairment of the right eye (Figure 1A). The fundus was normal. Laboratory tests showed elevated levels of lactate dehydrogenase (LDH) (318 U/L) and soluble interleukin-2 receptor (sIL2-R) (1280 U/mL). Cerebrospinal fluid analysis, including cytology, was normal. Body computed tomography (CT) showed splenomegaly. Brain magnetic resonance imaging showed abnormal diffuse signals in both orbital soft tissues with right-dominant, non-mass-like enhancements (Figure 1B and C). There were no lesions in the brain or paranasal sinuses. His visual acuity rapidly deteriorated to light perception within four days of admission. Biopsy of the orbital lesions was not performed because of the difficulty of approaching the orbit and the high surgical risk. Also, random skin biopsy and positron emission tomography/CT were not performed. We empirically administered intravenous methylprednisolone and antifungals, considering the possible etiology of autoimmune diseases or fungal infections, although the differential diagnoses were manifold and included neoplasms. His ocular symptoms partially recovered after every intravenous methylprednisolone treatment but continued to deteriorate shortly after treatment. Moreover, laboratory examination showed pancytopenia, and bone marrow examination revealed hemophagocytosis. He died of multiple organ failure 42 days after admission.

**Figure 2.**
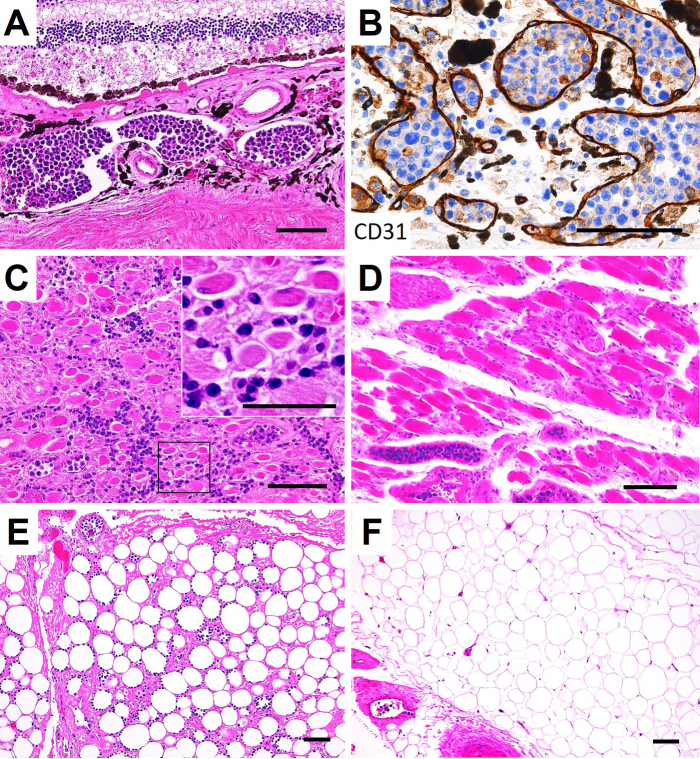
Histopathologic findings of the ocular adnexa of the right orbit. (A) Lymphoma cells proliferated within the lumina of small blood vessels in the choroid of the right eye (hematoxylin and eosin [H&E] staining). (B) Proliferation of lymphoma cells inside CD31-positive vascular endothelial cells with minimal extravascular invasion in the choroid of the right eye (CD31 immunohistochemical staining). (C) The extraocular muscles of the right eye showing atrophic muscle fibers and lymphoma cell invasion in the endomysium. A high-power view of the outlined area showed endomysial fibrosis with no evident necrotic or regenerating muscle fibers (inset) (H&E staining). (D) The extraocular muscles of the left eye showing non-atrophic fibers without extravascular invasion of lymphoma cells in the endomysium (H&E staining). (E) The right orbital adipose tissue showing lymphoma cell invasion (H&E staining). (F) The left orbital adipose tissue without extravascular invasion of lymphoma cells (H&E staining). Bars = (A-F) 100 μm; (inset in C) 50 μm.

**Figure 3.**
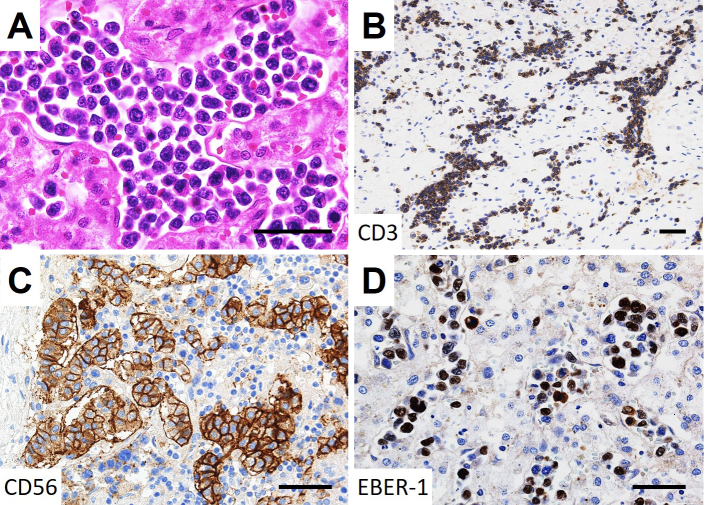
Histopathologic findings of the small nodule in the left adrenal gland. (A) Large lymphoma cells with irregular nuclei and scant cytoplasm proliferated inside the sinusoidal capillaries in the small nodule in the left adrenal gland (H&E staining). Immunohistochemical staining showed the lymphoma cells positive for CD3 (B), CD56 (C), and Epstein-Barr virus-encoded RNA 1 (EBER-1) (D). Bars = 50 μm.

On autopsy, lymphoma cells had proliferated profusely within the lumina of small blood vessels, in the choroid of both eyes (Figure 2A and B) and the extraocular muscles and adipose tissue of both orbits, with right dominance (Figure 2C, D, E and F), as well as the other examined organs (brain, heart, lung, intestine, pancreas, spleen, kidney, bladder, and bone marrow). No lymphoma cells were observed in the optic nerves. Minimal extravascular invasion of lymphoma cells was observed in the ocular adnexa (choroid, extraocular muscles, and adipose tissue) of the right orbit (Figure 2C and E), adipose tissue surrounding the lymph nodes and bone marrow. The only mass lesion was a small nodule (6 mm in diameter) in the left adrenal gland. An immunohistochemical study of the lymphoma cells showed positivity for CD3, CD56, and Epstein-Barr virus (EBV)-encoded RNA 1, consistent with natural killer/T-cell lymphoma (Figure 3). These lymphoma cells were positive for cytotoxic markers, such as granzyme B, perforin, and T-cell internal antigen-1, and negative for CD4, CD5, CD7, CD8, CD20, CD30, CD57, and CD79a. Polymerase chain reaction identified T-cell receptor (TCR)-β and TCR-δ gene rearrangements. These findings show that the patient was diagnosed postmortem with IVNKL.

## Discussion

Intravascular lymphoma is a rare subtype of extranodal non-Hodgkin lymphoma, and most cases are of B-cell lineage. To date, 25 cases of IVNKL have been documented ^(3), (4)^. The clinicopathological characteristics of IVNKL include occurrence in East Asian countries (12/25 cases), EBV positivity (20/24), and a fatal course (15/23). Because of the similarities in ethnicity, EBV status, and poor prognosis, as well as the immunophenotypic similarity of lymphoma cells between IVNKL and extranodal natural killer/T-cell lymphoma, nasal type (ENKLN), IVNKL may be a variant of ENKLN, differentiated by its intravascular nature ^(4)^. The dominant organs involved in IVNKL are the skin (23/25), central nervous system (8/25), bone marrow (2/25), kidneys (2/25), heart, lungs, spleen, liver, ileum, ovaries, and cervix (1/25 each). This case demonstrates that IVNKL can involve the ocular adnexa and manifest initially as ocular symptoms. Because of the patient’s atypical clinical features, including the ocular symptoms, the diagnosis was challenging, although his old age, weight loss, and elevated LDH and sIL2-R levels suggested lymphoma.

Based on the autopsy findings, we speculate that intravascular proliferation of lymphoma cells in the choroid might degrade visual acuity because vision loss was observed in a case of intravascular lymphoma of B-cell lineage with choroidal involvement ^(5)^. We also speculate that the atrophic fibers in the extraocular muscles of the patient’s right eye might be one cause of oculomotor impairment because intravascular lymphoma invasion of the muscles could induce a myopathic change ^(6)^.

In conclusion, IVNKL could involve the ocular adnexa. To ensure early diagnosis, even in patients who initially present with only ocular symptoms, IVNKL should be considered.

## Article Information

### Conflicts of Interest

None

### Author Contributions

N.U., D.Y., A.H., A.N., S.M., T.T., M.M., and H.M. were involved in the acquisition, analysis, or interpretation of data. N.U. drafted the manuscript. All authors contributed to the revision of the manuscript and approved the final version.

### Approval by Institutional Review Board (IRB)

The patient’s son signed informed consent forms for academic use of the data.

IRB Approval Code and Name of the Institution: not applicable
